# Evaluation of cerebral concussion using the SCAT-5 tool: translation into Brazilian Portuguese and cultural adaptation

**DOI:** 10.31744/einstein_journal/2026AO2124

**Published:** 2026-06-23

**Authors:** Ana Camila de Castro Gandolfi, Patrícia Logullo, Cristina Casagrande Miranda Teixeira, Moisés Cohen, Rachel Riera

**Affiliations:** 1 Universidade Federal de São Paulo Escola Paulista de Medicina Neurology and Neurosurgery Department São Paulo SP Brazil Neurology and Neurosurgery Department, Escola Paulista de Medicina, Universidade Federal de São Paulo, São Paulo, SP, Brazil.; 2 Independent Researcher Oxford United Kingdom Independent Researcher, Oxford, United Kingdom.; 3 Universidade Federal de São Paulo Escola Paulista de Medicina Orthopaedics Department São Paulo SP Brazil Orthopaedics Department, Escola Paulista de Medicina, Universidade Federal de São Paulo, São Paulo, SP, Brazil.; 4 Universidade Federal de São Paulo Escola Paulista de Medicina Medicine Department São Paulo SP Brazil Medicine Department, Escola Paulista de Medicina, Universidade Federal de São Paulo, São Paulo, SP, Brazil.

**Keywords:** Brain concussion, Brain injuries, Athletic injuries, Surveys and questionnaires

## Abstract

**Objective:**

This study aimed to translate and culturally adapt the Sports Concussion Assessment Tool version 5 (SCAT-5) in Brazilian Portuguese for the follow-up of patients diagnosed with cerebral concussion.

**Methods:**

The components of the SCAT-5, which includes an assessment of level of consciousness using the Glasgow Coma Scale, were translated from English to Brazilian Portuguese, back-translated, and evaluated by an expert committee. The final version was tested twice, with a 2-week interval between assessments, in 36 athletes admitted to the cerebral concussion outpatient clinic with concussion. Statistical analyses were subsequently performed using prevalence-adjusted and bias-adjusted kappa to assess the degree of agreement between the first and second tests.

**Results:**

The athletes were predominantly rugby players (23/36) and female (23/36), and none were in the acute phase of concussion. Their ages ranged from 18 to 45 years, and the median time from the last concussion to assessment was 104 days. A significant difference was observed between the first and second tests for three variables evaluated: "total number of symptoms," "balance test [standing on one foot]" and "total balance score," with an increase in the number of symptoms and a decrease in balance scores between tests, possibly indicating worsening symptoms or increased awareness about symptoms. The agreement between the first and second assessments was deemed moderate only for "irritability" and good or very good for all other variables.

**Conclusion:**

The SCAT-5 was successfully translated and culturally adapted for use in Brazilian patients. This tool will contribute to improving and standardizing the healthcare of patients with cerebral concussion in Brazil, improving medical care, and guiding medical staff toward orienting patients to a gradual and adequate return to sports-related activities following concussion.

## In Brief

We translated and culturally adapted the Sports Concussion Assessment Tool version 5 (SCAT-5) under the supervision of an expert group. This validated tool will allow standardized evaluation in sports emergency care.

## Highlights

■ The SCAT-5 is a questionnaire for the follow-up of patients diagnosed with cerebral concussion.
■ The SCAT-5 was translated from English to Brazilian Portuguese, back-translated, and evaluated by an expert committee. The final version was tested twice on athletes admitted to the cerebral concussion in Sports outpatient clinic with concussion.
■ The SCAT-5 was successfully translated and culturally adapted for use in Brazilian patients. The agreement between the first and second tests was moderate for "irritability" and good or very good for all other variables.
■ The Brazilian Portuguese SCAT-5 can contribute to improving and standardizing the healthcare of patients with cerebral concussion in Brazil.

## Graphical abstract

**Figure:**
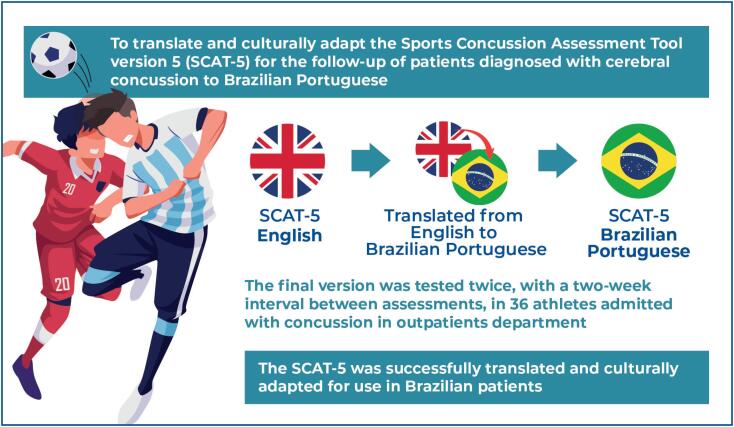

## INTRODUCTION

Cerebral concussion is a traumatic event in the cephalic region that leads to transient alterations in brain function. It is most often self-limiting,^(1)^ meaning that neurological impairment following concussion generally lasts for only a brief period, the signs and symptoms resolve spontaneously, and no identifiable structural damage was observed on conventional imaging.^(2)^ Nevertheless, more than 30% of patients still report symptoms up to 1 year after trauma.^(3)^ Mild head trauma is the most common cause of concussion. Brain concussions generally occur through rapid acceleration, deceleration, and rotational forces, which cause diffuse biochemical stress, leading to transient, mostly reversible alterations in neuronal function.^(4)^

Concussions are the most common form of traumatic brain injury, accounting for up to 9% of all sports-related injuries.^(5,6)^ In Europe, 235 of every 100,000 patients admitted to hospitals have traumatic brain injuries, of which 80% are considered mild.^(6,7)^ In the USA, it is estimated that 1.58 million people experience traumatic brain injuries annually, of which 75%–90% are mild,^(6)^ and head trauma is the main cause of disability in young adults in the USA. The importance of concussion lies mainly in its relationship with second-impact syndrome^(8-10)^ and chronic traumatic encephalopathy.^(11)^ Second impact syndrome is a condition caused by new trauma to the brain before it has fully recovered from an initial concussion, possibly leading to disastrous results and even death.^(9,10)^ The etiology of this syndrome is not yet fully understood; however, new trauma in a not completely recovered brain is assumed to trigger the loss of cerebral autoregulation, leading to vasoplegia associated with vasodilation of cerebral capillaries and secondary edema.^(12)^ Chronic traumatic encephalopathy is a progressive neurodegenerative syndrome caused by multiple direct impacts to the head and the resulting transmission of acceleration/deceleration forces to the brain. The clinical presentation typically appears after a long latency period and can include psychiatric, behavioral, and cognitive changes with or without sensory or motor impairment.^(13)^

With the growing concern related to the repercussions of cerebral concussion, in November 2001, the 1^st^ Symposium on Concussion in Sport discussed the diagnosis and follow-up of patients with concussion, subsequently instituting a care protocol.^(14)^ Concussion is generally diagnosed clinically; in the case of sports injury, it is diagnosed by medical evaluation in the field using a simplified neurological evaluation performed promptly after any trauma that may have caused concussion. In 2004, at the 2^nd^ Symposium on Concussion in Sport, the first version of the Sports Concussion Assessment Tool (SCAT) was created.^(15)^ Throughout the subsequent Symposia on Concussion in Sport, the SCAT underwent modifications, yielding the SCAT-5.^(16)^ The SCAT is not intended to be a diagnostic tool but rather an instrument to facilitate follow-up in athletes with recent or previous concussions, assisting physicians in recommending when athletes can return to activity.^(16)^

The SCAT-5 is a compilation of tools widely used in medicine to evaluate memory and level of consciousness (using the Glasgow Coma Scale [GCS]), signs indicating a possible alteration in the cervical spine, neurological screening, and assessments of cognition, concentration, and balance. For greater accuracy, every athlete with cerebral concussion should undergo SCAT evaluation at the first assessment to establish a baseline.^(17,18)^ The tool investigates the history of previous injuries as well as their current signs and symptoms. No Portuguese tool has been designed to assess athletes with concussions. This can affect the care of patients with head trauma, making it necessary to translate and culturally adapt the SCAT-5.

## OBJECTIVE

This study aimed to translate and culturally adapt the Sports Concussion Assessment Tool version 5 (SCAT-5) for patients diagnosed with cerebral concussion who are native speakers of Brazilian Portuguese.

## METHODS

### Study design

The translation and cultural adaptation study was conducted at a public university sports trauma clinic in São Paulo, Brazil. It included the translation of the SCAT-5 tool from English to Portuguese, in addition to testing the cultural validity of the translated version among athletes who had experienced head trauma. The study protocol was approved by the Independent Ethics Committee of the *Universidade Federal de São Paulo,* CAAE:62592516.1.0000.5505; #1.865.130. All participants provided written informed consent. The translation of the SCAT-5 into Brazilian Portuguese, the primary language spoken in Brazil, was performed between 2018 and 2020, following the receipt of official authorization by the corresponding author of the SCAT-5.

### Translation procedures

Overall, the methodologies proposed by Guillemin et al.^(19)^ and Beaton et al.^(20)^ were used, including translation, evaluation by an expert panel, back-translation, and approval by the original authors. The translated tool was subsequently tested and retested among athletes who had suffered brain trauma, allowing for within-participant comparisons. The steps of this study were:

Step 1: Translation. The initial SCAT-5 translation into Portuguese was performed independently by three native speakers of Brazilian Portuguese. First, the T1 translation was conducted by a physician with prior knowledge of the SCAT-5. The T2 translation was performed by a journalist informed about the study objectives but who was unfamiliar with the tool. Finally, the T3 translation was conducted by an engineer fluent in English who was not familiar with the tool or study subject. The three translators had no contact with each other during the study and were instructed to not discuss the topic with others.

Step 2: Comparison: The three versions (T1, T2, and T3) were compared using an online platform (Google Forms; Google LLC, Mountain View, CA, USA). An eight-person committee was assembled and asked to choose the best or most appropriate translation of each item. All committee members were English-fluent Brazilian citizens and native speakers of Brazilian Portuguese. Of the eight members, four were physicians (trauma surgeon, rheumatologist, obstetric/gynecologist resident, and health technology assessment specialist). The other members included a psychologist, a physical trainer, and two businesspeople (one with a health sector background). Committee members were free to comment on and suggest other translation alternatives when none of the three options seemed acceptable. They could also ask questions via email but had no contact with each other or knowledge of the other committee members to ensure independent voting.

Item translations with at least five out of eight votes were considered acceptable. The items that did not reach this threshold were subjected to a second round of voting. Adjustments were made to address any issues highlighted in committee comments. The final approved version, T4, was submitted for back-translation.

Step 3: Back-translation into English: The T4 version, approved by the committee, was translated from Portuguese back into English by two professional translators who are native English speakers with a good knowledge of Brazilian Portuguese. Neither of the translators had knowledge of the study objectives. Each back-translated version was kept separate and are referred to as T5 and T6, respectively.

Step 4: Final version: A final comparison of the three versions, T4 (in Portuguese), and T5 and T6 (in English), allowed for resolution of any differences. The objective of this step was to solve issues with semantics (meaning) and make them more understandable to Brazilian Portuguese speakers. In addition to semantic equivalence, aspects of idiomatic equivalence (such as expressions that are impossible to translate, for which new idioms must be created) and the reality of Brazilians and their experiences (which may not be normal in English-speaking environments) were considered. The final Portuguese version, T7, was developed based on T4 and modified based on T5 and T6.

Step 5: Test application: T7 was administered to 36 patients with a history of cerebral concussion who underwent evaluation at the University's Cerebral Concussion in Sports Outpatient Clinic. Administration was performed by physicians familiar with the original tool (in English) who read the recommendations for the application of the T7 version.

All patients were evaluated twice, with a 2-week period between each assessment. The main objective of testing was to check whether the participants reported problems regarding a lack of understanding of the questionnaire.^(19,21,22)^ If a patient had difficulty understanding a question, the committee reconvened to discuss additional changes. The goal of this sequence of tests was for each participant to understand at least 90% of the questions.

Step 6: Testing and retesting: Responses to the first and second assessments (test and retest) were compared. The SCAT sections were considered to be independent, with no interrelationships between them. As such, the analysis of each item was performed individually. Only items with numerical responses were included in this analysis, as some SCAT-5 items have open-ended rather than numerical answers.^(23,24)^

### Statistical analysis

Statistical analyses were performed by comparing the patients’ responses in the first and second assessments. Because answers were highly concentrated in the same categories, the agreement was evaluated using prevalence and bias-adjusted kappa (PABAK).^(25)^ This approach was also used to address agreement for: GCS items; symptom scale items (with each symptom analyzed separately); total number of symptoms; orientation; memory; digits; balance test; and delayed memory. The degree of agreement was used for analysis (Table 1). Statistical analyses were performed using IBM SPSS Statistics version 23 (IBM Corp., Armonk, NY, USA).

**Table 1:** Classification by kappa and PABAK coefficient^(25)^

*Kappa* coefficient	Interpretation of the correlation
0.8 to 1	Very good
0.61 to 0.8	Good
0.41 to 0.6	Moderate
0.21 to 0.4	Reasonable
<0.2	Poor

PABAK: prevalence and bias-adjusted kappa.

## RESULTS

### Translation

Versions T1, T2, and T3 were generated by translating from the original language (English) into Brazilian Portuguese. In one of the sentences, one translator made more than one translation suggestion; thus, both options were added to the Google Forms questionnaire so that the committee could vote on one of the four options. When sentences/expressions were translated in the same way by all translators, they were not included in the Google Forms questionnaire. For 13 questions, two translators proposed the same translation, leaving only two alternatives in the Google Forms questionnaire.

For five questions, none of the options obtained five votes, necessitating a second round of voting. The two alternatives with the highest number of votes were selected in the second round, and a tiebreaker was not required in this round. After the second round of voting, prior to back-translation, the committee members were allowed to provide suggestions and ask questions about the context of the test via e-mail. One committee member indicated the inclusion of the terms "decerebration" and "decortication" in the GCS instead of the terms "extension in response to pain" and "abnormal flexion in response to pain," respectively, as these two terms are well known among Brazilian physicians who apply the GCS. The addition was made separately from the translation of the term, indicated by slashes ("/"), as follows: most voted translation of "extension in response to pain"/decerebration.

Another issue raised by the committee was the presence of a translation error comprising a false cognate (a word that is phonetically and graphically similar in both languages but has different meanings)^(26)^ among the alternatives. In this case, one alternative translated the English term "severe" to Portuguese as "severo" (which in Portuguese means strict, rigid), when the correct translation should be the Portuguese word "grave" The research team explained to the committee that, according to the methodology adopted, interfering with the translation alternatives would not be possible at this voting phase but could occur in the evaluation of the final version, if necessary. However, no adjustment was deemed necessary, as the erroneous translation of "severe" was chosen by only one committee member.

### Administration

The test was administered to athletes who had previously sought services for cerebral concussion. The inclusion criterion was the diagnosis of at least one concussion in adulthood. However, none of the evaluated patients were in the acute post-concussion phase. For the purpose of this study, we considered the acute post-concussion phase to be the first 24–48 h after head trauma, according to the guidelines of the 5^th^ International Conference on Concussion in Sport.^(16)^ The athlete who reported the most recent concussion had experienced trauma 3 days prior to the assessment.

Among the 36 evaluated patients, a predominance of women (23 women, 13 men [63.8% women]) and rugby athletes (23/36 [63.8%]) was noted. Participant ages ranged from 18 to 45 years, with a mean of 25.92 years (standard deviation, 5.26). The time from the last concussion to assessment ranged from 3 to 1,385 days, with a mean of 235.4 days and a median of 104 days. The educational level of the sample ranged from elementary school to higher education: 5% had completed elementary school, 64% had high school diplomas, and 31% had college degrees.

Among the most frequent symptoms were "fatigue or low energy" (20/36 patients [55.5%]), "difficulty remembering" (20/36 patients [55.5%]), and "difficulty concentrating" (19/36 patients [52.7%]). The emotional symptoms of "more emotional" and "nervous or anxious" were also quite frequent, both appearing in 15 patients (41.6%).

The GCS assessment showed no variability in the results, with a score of 15 in all patients. No statistical analysis was performed on the GCS data because no variability was observed between patients or between the same patients in each evaluation.

**Figure 1:**
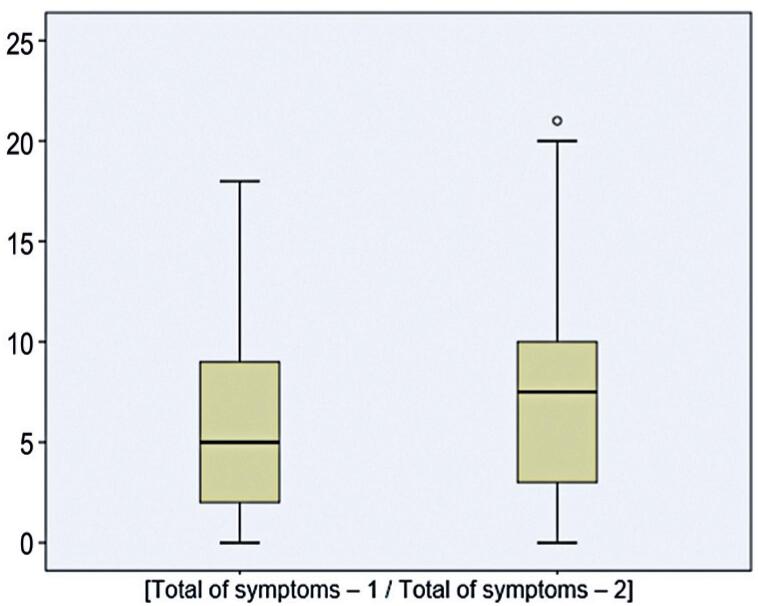
Variation in total of symptoms between first and second assessments (maximum, average and minimum)

**Figure 2:**
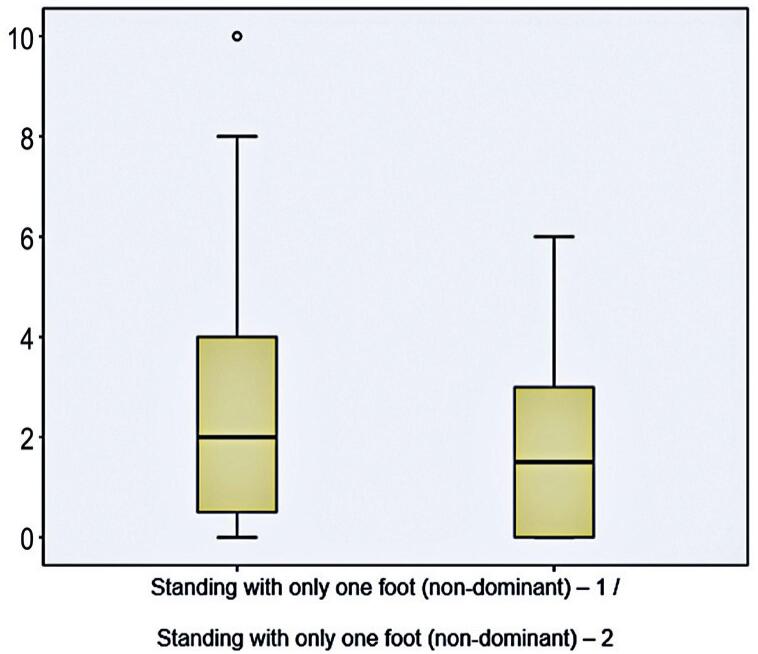
Difference between scores (number of errors) in the first and the second test for standing with only one foot

**Figure 3:**
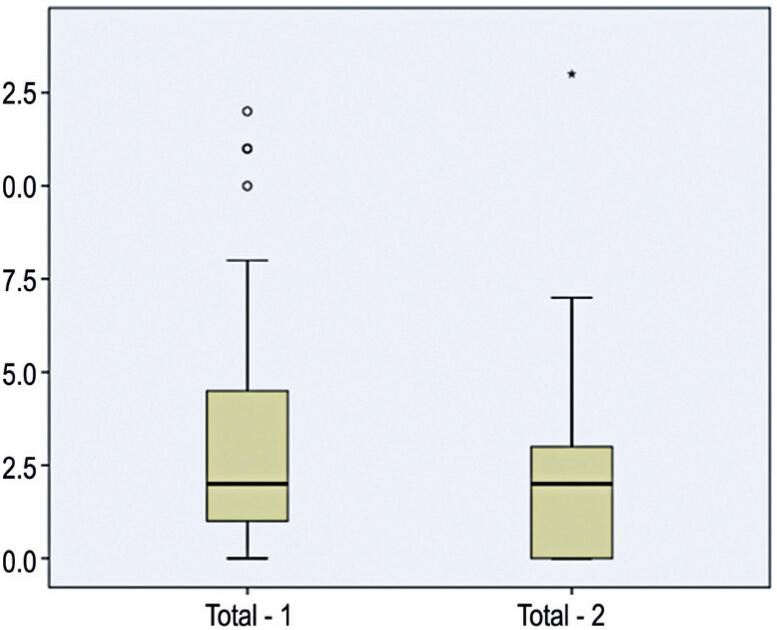
Difference between the total scores (number of errors) on the first and second tests

A significant difference was observed between the first and second test only for the variables "total number of symptoms" (Table 2), "balance test – standing with only one foot" (Table 3) and "total balance score" (Table 4). The data showed an increase in the total number of symptoms and a decrease in balance scores (Figures 1 to 3).

**Table 2:** Symptoms – total

Total of symptoms	1	2	p value
Mean (SD)	6.22 (5.32)	7.64 (5.47)	0.0336
Median	5	7.5	

SD: standard deviation.

**Table 3:** Balance test – Standing with only one foot (non-dominant)

Standing with only one foot (non-dominant)	1	2	p value
Mean (SD)	2.67 (2.64)	1.72 (1.73)	0.0436
Median	2	1.5	
Minimum–maximum	0–10	0–6	
Total of patients	36	36	

SD: standard deviation.

**Table 4:** Balance test – Total score

Total score	1	2	p value
Mean (SD)	3.47 (3.39)	2.39 (2.81)	0.0430
Median	2	2	
Minimum–maximum	0–12	0–13	
Total of patients	36	36	

SD: standard deviation.

The degree of agreement was deemed moderate only for "irritability" and good or very good for all other variables.

## DISCUSSION

The predominance of women in the sample in this study reflects real-world situations related to concussion in sports.^(27,28)^ A higher prevalence of concussion was observed among women, and the reasons for this are not well understood; however, two factors seem to play an important role. First, women have less muscle mass in the cervical region, meaning they have less cushioning against head trauma. Second, estrogen plays a role in cerebral blood flow.^(29)^ The preponderance of rugby players in the sample is likely related to the body-to-body contact nature of this sport, which leads to a higher incidence of concussion.^(29)^ The predominance of women in our sample may be because women are more likely to seek medical assistance for mild symptoms than men.

The total number of present symptoms (Table 1S, Supplementary Material) increased from the first to the second assessment. No patients reported a new concussion between assessments; the increase in the total number of symptoms could be explained by clinical worsening after the initial concussion or by greater patient perception regarding relevant symptoms due to the first assessment. Test–retest variability for the SCAT-5 has not been reported in the literature. No significant differences were observed in the Orientation, Memory, Digits, or Delayed Memory items between the two assessments. This reinforces the idea that, ideally, comparisons should be performed within patients; that is, whenever possible, a baseline assessment should be performed so that, in the case of concussion, within-patient comparisons can be made. In terms of Balance, no significant differences were noted in the items "standing with both feet" and "tandem" (one foot in front of the other); however, a difference was observed in the balance test total score and the item "balance test – standing with only one foot." A significant difference was also found in balance scores between the two assessments. As the balance score was determined by the number of errors, an improvement in the patients’ balance could be inferred between assessments, either due to learning or clinical improvement.

The relatively small sample size of 36 patients may be considered a limitation of our study; however, the existing literature supports the inclusion of fewer than 30 participants in studies focusing on translation and cultural adaptation. It could be argued that differences could exist between assessments conducted during the acute and chronic phases of concussion. Nevertheless, the present findings reflect a highly heterogeneous population in terms of the time between traumatic brain injury and assessment, which is representative of the patient profiles typically encountered in outpatient clinics. Finally, the widespread use of the translated tool in Brazil may provide additional data and reveal specific characteristics of Brazilian athletes beyond the scope of this study.

Although English use is widespread in Brazil, it cannot be considered a true second language. Even in places where English is a common second language, the SCAT-5 has been translated and culturally adapted into non-English languages because the use of a patient's non-native language can lead to misinterpretations, potentially compromising the quality of care.^(29)^ Furthermore, the use of direct translation into another language without cultural considerations can make a test less reliable. In the Mandarin Chinese translation of the SCAT-3, for example,^(30)^ the item " name the months in reverse order" was adapted and changed to a task in which patients subtract 3 from 100 repeatedly. This is because months in this language are named using their number (i.e., "Month 1", "Month 2", etc.") and not using specific names; therefore, it is likely easier for patients to name the months in reverse order.^(29,30)^

The SCAT-5 has been translated into other languages to facilitate its use among athletes and sports associations. In its traumatic brain injury guidelines, the World Rugby Confederation recommends the use of the SCAT-5 for patient follow-up.^(31)^ The SCAT has also been proposed as a means of unifying the follow-up of patients with concussion worldwide; therefore, its use is supported by the *Fédération Internationale de Football Association*, the International Ice Hockey Federation, World Rugby, the *Fédération Equestre Internationale*, and the International Olympic Committee.^(16)^ In addition, by using a recognized method for translation and cultural adaptation of the tool, future studies can be conducted using the SCAT-5 in Brazilian Portuguese, which would be of great value in Brazilian settings in which epidemiological data regarding concussion in sports and post-concussion syndrome are still lacking. In addition, the translation method used in this study can be applied to translate the SCAT-5 into other languages, which could improve the integration of concussion data worldwide.

## CONCLUSION

In this study, the SCAT-5 was successfully translated into Brazilian Portuguese and culturally adapted, yielding an adequate tool for the follow-up of patients with cerebral concussion and post-concussion syndrome, which could facilitate better and more complete care.
