# Supplementary material for: Evaluation of cerebral concussion using the SCAT-5 tool: translation into Brazilian Portuguese and cultural adaptation
Source: Einstein (Sao Paulo). 2026 Jun 23;24:eAO2124. doi: 10.31744/einstein_journal/2026AO2124 (PMC13399293; doi:10.31744/einstein_journal/2026AO2124)
Supplement: Supplementary Material [file 2317-6385-eins-24-eAO2124-suppl01.pdf]

SUPPLEMENTARY MATERIAL

Evaluation of cerebral concussion using the SCAT-5 tool: translation into Brazilian Portuguese and cultural adaptation

Ana Camila de Castro Gandolfi, Patrícia Logullo, Cristina Casagrande Miranda Teixeira, Moisés Cohen, Rachel Riera

DOI: 10.31744/einstein_journal/2026AO2124

Table 1S. SCAT-5 symptoms and their frequency, agreement rates, and p values

	1st Assessment							2nd Assessment							Agreemet			
	0	1	2	3	4	5	6	0	1	2	3	4	5	6	p-Value	Kappa	PABAK	Interpretation
Headache	0	23	9	2	1	1	0	0	23	8	4	1	0	0	0,8912	0,21	0,76	Good
Pressure in head	0	31	2	2	1	0	0	0	30	2	3	1	0	0	0,5037	0,19	0,81	Very Good
Neck pain	0	27	6	1	2	0	0	0	25	6	4	1	0	0	0,5249	0,32	0,69	Good
Nausea or vomiting	0	34	2	0	0	0	0	0	31	3	1	1	0	0	0,8088	0,08	0,85	Very Good
Dizziness	0	31	3	1	1	0	0	0	28	6	1	1	0	0	0,1991	0,29	0,8	Very Good
Visão embaçada	0	29	4	3	0	0	0	0	28	6	2	0	0	0	0,881	0,37	0,72	Good
Balance problems	0	27	6	2	1	0	0	0	25	7	1	3	0	0	0,3262	0,44	0,8	Very Good
Sensitivity to light	0	29	4	0	2	1	0	0	22	9	2	2	1	0	0,7717	0,18	0,76	Good
Sensitivity to noise	0	31	3	1	0	1	0	0	26	5	3	2	0	0	0,7907	0,08	0,72	Good
Feeling slowed down	0	28	4	3	0	1	0	0	27	5	2	2	0	0	0,9996	0,37	0,85	Very Good
Feeling like "in a fog"	0	34	1	0	1	0	0	0	33	2	1	0	0	0	0,8866	-0,05	0,85	Very Good
"Don't feel right"	0	29	3	1	3	0	0	0	26	7	2	0	0	1	0,8424	0,38	0,85	Very Good
Difficulty concentrating	0	17	11	4	2	2	0	0	16	10	6	3	0	1	0,9772	0,43	0,81	Very Good
Difficulty remembering	0	15	9	7	3	1	1	0	11	16	3	4	2	0	0,9545	0,21	0,7	Good
Fatigue or low energy	0	15	11	5	4	1	0	0	17	8	8	3	0	0	0,9559	0,48	0,75	Good
Confusion	0	30	4	1	1	0	0	0	30	2	3	1	0	0	0,6746	0,16	0,78	Good
Drowsiness	0	18	9	7	1	1	0	0	20	10	6	0	0	0	0,9407	0,24	0,67	Good
More emotional	0	24	6	3	1	1	1	0	18	12	4	1	1	0	0,9685	0,35	0,77	Good
Irritability	0	24	4	8	0	0	0	0	16	16	2	2	0	0	0,0742	0,09	0,56	Moderate
Sadness	0	30	3	2	1	0	0	0	29	6	1	0	0	0	0,8494	0,11	0,74	Good
Nervous or Anxious	0	19	6	5	5	1	0	0	14	11	8	2	0	1	0,7041	0,25	0,68	Good
Trouble falling asleep	0	23	3	2	5	2	1	0	22	7	5	1	1	0	0,7758	0,14	0,63	Good
